# A Systematic Review of Adherence to Immunosuppression among Pediatric Heart Transplant Patients

**DOI:** 10.3390/jcdd9050165

**Published:** 2022-05-23

**Authors:** Keira Nassetta, Tasmeen Hussain, Katheryn Gambetta, Kevin Le, Linda C. O’Dwyer, Sherif M. Badawy

**Affiliations:** 1 Department of Pediatrics, Ann and Robert H. Lurie Children’s Hospital of Chicago, 225 E. Chicago Avenue, Chicago, IL 60611, USA; knassetta@luriechildrens.org; 2 Department of Internal Medicine, Northwestern University McGaw Medical Center, 251 E. Huron St., Ste. 16-738, Chicago, IL 60611, USA; tasmeen.hussain@northwestern.edu; 3 Division of Cardiology, Ann and Robert H. Lurie Children’s Hospital of Chicago, 225 E. Chicago Avenue, Chicago, IL 60611, USA; kegambetta@luriechildrens.org; 4 Department of Pharmacy, Ann and Robert H. Lurie Children’s Hospital of Chicago, 225 E. Chicago Avenue, Chicago, IL 60611, USA; kle@luriechildrens.org; 5 Galter Health Sciences Library & Learning Center, Northwestern University Feinberg School of Medicine, 320 E. Superior Street, Chicago, IL 60611, USA; l-odwyer@northwestern.edu; 6 Division of Hematology, Oncology, and Stem Cell Transplant, Ann and Robert H. Lurie Children’s Hospital of Chicago, 225 E. Chicago Avenue, Chicago, IL 60611, USA; 7 Department of Pediatrics, Northwestern University Feinberg School of Medicine, 225 E. Chicago Ave., Chicago, IL 60611, USA

**Keywords:** heart transplant, adherence, compliance, pediatric, immunosuppression, rejection

## Abstract

After pediatric heart transplant, commitment to lifelong immunosuppression is crucial to maintaining graft health. However, a review of the current literature surrounding adherence to immunosuppression in pediatric heart transplant patients is lacking. This systematic review aims to summarize the current landscape of adherence to immunosuppression in pediatric heart transplant patients. We conducted searches in PubMed MEDLINE, Embase, CENTRAL register of Controlled Trials (Wiley), and Scopus, from inception to March 2020. Studies were eligible if they outlined an aspect of adherence to immunosuppression and the measurement of adherence was performed with an objective or otherwise validated measure of adherence (e.g., drug levels, adherence questionnaires). The titles/abstracts of 880 articles were reviewed. After initial screening, 106 articles underwent full text review. As such, 14 articles were included in the final review. Baseline adherence estimates varied greatly, with most values between 40% and 70%. Nonadherence to immunosuppression is associated with worse outcomes (rejection, hospitalization, mortality), impaired quality of life, and mental health concerns in pediatric heart transplant patients. As nonadherence to immunosuppression is common and associated with worse outcomes, there is a need for further development and evaluation of interventions in this space.

## 1. Introduction

Pediatric heart transplantation is the standard of care for select patients with end-stage heart disease [1,2]. The primary indication for transplant differs by age, with congenital heart disease being most common in infants (57%) and cardiomyopathy being most common in older children (43% in children aged 1–10 years and 53% in children aged 11–17 years) [3].

After pediatric heart transplant (HTx), commitment to lifelong immunosuppression is necessary to maintain graft health. Most post-transplant immunosuppression regimens include a calcineurin inhibitor (CNI) and an antiproliferative agent [2,4]. Adequate adherence to these immunosuppressive medications is essential to preventing poor outcomes [5]. Estimates for rates of nonadherence within the pediatric population are wide ranging, likely due to the lack of standardization in adherence reporting [6]. Additionally, assessing adherence can be difficult due to the subjective nature of self-report. A review of the current literature surrounding adherence to immunosuppression in pediatric heart transplant patients is lacking.

In this manuscript, we evaluated measures of adherence, impact of nonadherence, and interventions to improve adherence outlined in the literature. Our objective was to review and describe the current landscape of immunosuppression adherence in pediatric heart transplant patients by identifying adherence rates and related factors, as well as proposed interventions for improving adherence in this population.

## 2. Methods

### 2.1. Study Design

The authors followed all guidelines for the Preferred Reporting Items for Systematic Reviews and Meta-Analyses (PRISMA) statement [7].

### 2.2. Search Strategy

We conducted a systematic review of studies on adherence to immunosuppression in adult and pediatric HTx patients. A research librarian (LO) was responsible for a full literature search. Searches were conducted in PubMed MEDLINE, Embase, CENTRAL register of Controlled Trials (Wiley), and Scopus, from inception to March 2020, using search strategies that were collaboratively developed by the authors. The search was employed in PubMed using a combination of MeSH terms for heart transplantation, compliance, and adherence. The same terms were then used with the other databases. Search strategies can be found in Appendix A. No date limits were used. There were only English articles included in the review. TH also hand searched the bibliographies of relevant review articles and the included articles for additional references. Articles were reviewed in Rayyan by two independent reviewers and discussed to reach consensus.

### 2.3. Eligibility Criteria

Studies were eligible if they outlined an aspect of immunosuppression adherence in HTx patients, including but not limited to measurements of adherence, outcomes associated with nonadherence, and strategies to improve adherence. Studies that included children and adolescent patients were eligible. Studies involving multiple types of solid-organ transplants were included, but the HTx cohort had to contain at least 10 participants and the HTx group data had to be separately reported. Included studies also utilized an objective or otherwise validated measure of adherence (e.g., drug levels or adherence questionnaires). Studies were excluded if adherence was measured with a non-validated measure (e.g., physician report). Studies could be a prospective, observational, cross-sectional survey, or randomized clinical trial.

### 2.4. Study Selection and Data Extraction

Two authors (TH and SB) independently screened the articles for inclusion and subsequently reviewed the full text of the included articles. Discordant assessments were resolved by discussion between the reviewers to reach consensus. Data extraction was standardized to include population, type of article, study design, immunosuppression used, intervention (if any), measure of adherence, duration, number of HTx participants, participant age, study attrition rate, and main outcomes.

### 2.5. Assessment of Risk of Bias

Bias was evaluated by two independent reviewers (TH and KN). Reviews utilized the Newcastle–Ottawa for cohort studies [8], a modified version of the Newcastle–Ottawa by Modesti et al. [9] for cross-sectional studies, and Version 2 of the Cochrane risk-of-bias tool (RoB 2) [10] for randomized trials. Disagreements were resolved by discussion between reviewers to reach consensus.

### 2.6. Data Synthesis

Data were expected to be heterogenous. If sufficient homogeneity was found in outcomes, a meta-analysis or effect size analysis was considered.

## 3. Results

### 3.1. Literature Search

**Figure 1:**
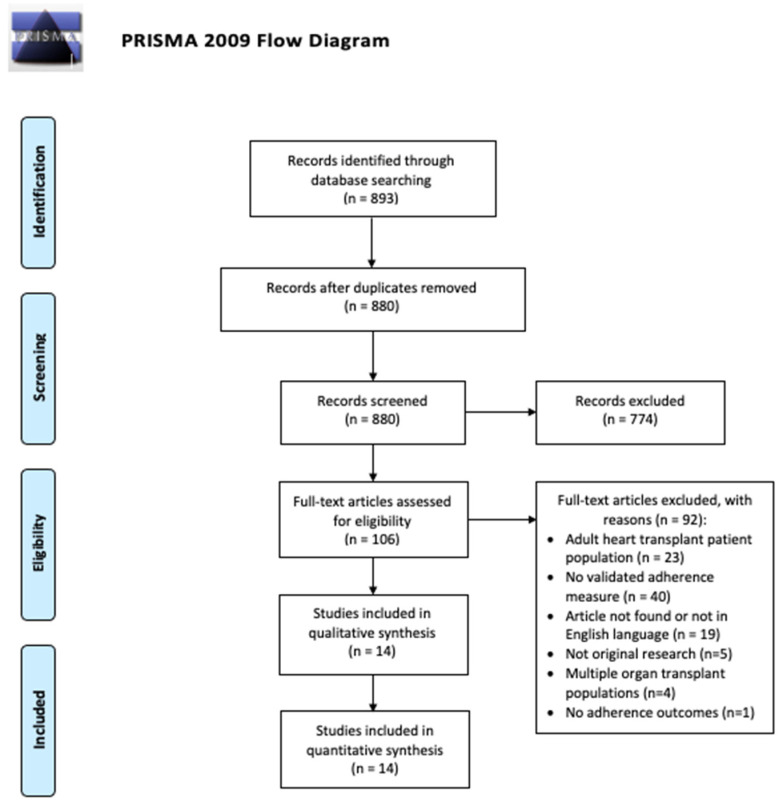
PRISMA Flow Diagram for the included studies.

The titles/abstracts of 880 articles published before March 2020 were reviewed and 774 articles were excluded in the initial screen, leaving 106 articles for full-text review. Ninety-two articles were excluded, and 14 were included in the final review. The study flowchart and further reasoning for article inclusion/exclusion are outlined in the PRISMA flow diagram (Figure 1).

### 3.2. Study Characteristics

Study characteristics, including study design, are outlined in Table 1. Studies were published between 1998 and 2019. Eleven studies were conducted in the USA [6,11,12,13,14,15,16,17,18,19,20] and three studies in Europe [21,22,23]. Study types included cross sectional (seven studies), cohort (six studies), and one randomized trial. The median number of pediatric heart transplant patients was 32, with a range of 12–138. Studies were looked at collectively to determine measures and rates of nonadherence. Studies were then divided into three themes: (1) CNI levels as a marker for nonadherence and correlation to poor outcomes (four studies), (2) impact of nonadherence on quality of life and mental health (seven studies), and (3) the effect of transition programs on adherence (three studies).

### 3.3. Measures and Rates of Nonadherence

**Figure 2:**
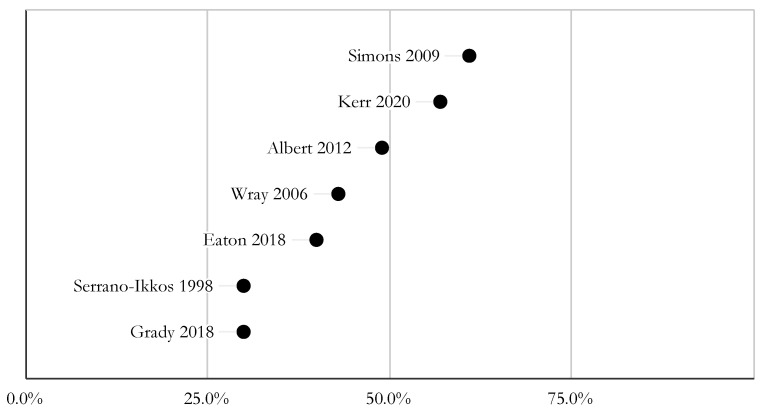
Reported non-adherence rates.

Studies used a variety of subjective (e.g., self-report) and objective (e.g., drug levels) measures. The reported rates of adherence varied greatly. In five studies, the nonadherence rate ranged from 40 to 60% [6,13,17,21,23]. Grady et al. (2018) found that approximately 70% of participants had CNI levels within the target range [16], and Serrano-Ikkos et al. (1998) found 69.8% of patients had good adherence, based on CNI levels and self-reported data [22]. In two studies, approximately 20% of patients reported at least one late or missing dose of immunosuppression medication in the last week [13,18], and 28% of caregivers reported that their adolescent/young adult (AYA) took one or more doses of antirejection medications late in the past week [13]. Figure 2 is a graphical representation of reported nonadherence rates, as described above. Table 2 outlines the different adherence measures used.

### 3.4. CNI Levels as a Marker for Nonadherence and Poor Outcomes

Table 3 outlines the four studies that included immunosuppressant levels as a marker for nonadherence and poor outcomes. Immunosuppression variability is associated with hospitalization [6,14], rejection [6,14,19], and mortality [6,14]. More specifically, Kerr et al. (2020) looked at rejection risk following subtherapeutic CNI levels. The risk of rejection increased 6.9-fold in the 2 weeks following a subtherapeutic level, and increased 6.1-fold in the 3 months after presenting with a subtherapeutic level (as compared to time period after a therapeutic level) [17]. Further, 22% required treatment for rejection within 3 months of a subtherapeutic level [17]. When looking at self-reported nonadherence, mortality was significantly correlated with adolescent reports of missed doses [6]. In contrast, Ringewald et al. (2001) did not find an association between self-reported nonadherence and abnormal CSA level at admission for rejection. Notably, two-thirds of the patients with late rejection (11 out of 15) admitted to nonadherence [19].

### 3.5. Impact of Nonadherence on Quality of Life and Mental Health

Table 4 outlines the seven studies that included the impact of nonadherence on quality of life and mental health. Some studies found that nonadherence was associated with patient mental health concerns on child and/or caregiver self-report tools [12,23], and poor quality of life scores further correlated with rejection episodes [12]. In two studies, specific psychiatric comorbidities, such as anxiety [18] and depression [21], were found to correlate with nonadherence. In contrast, two studies found that there were no associations between mental illness in the child and nonadherence [13,22], and that caregiver emotional distress did not seem to correlate with missed doses [13]. These findings may be explained by low rates of reported nonadherence, and an overall lower prevalence of adherence problems in the HTx group, as compared to other solid-organ transplant groups in the study [13].

### 3.6. The Effect of Transition Programs on Adherence

Table 5 outlines the two studies discussing transition from pediatric to adult health care. Anton et al. (2019) utilized a two-year structured transition program consisting of seven two-hour sessions to improve patients’ overall medical knowledge, medication adherence, readiness to transition, and parental perceptions of child’s readiness to transition [11]. They found a statistically significant decrease in percentage of CNI levels out of range prior to beginning the transition program and after completing the transition program [11]. In addition to improving immunosuppression adherence, the program also enhanced overall patient medical and medication knowledge, which may prevent lapses in medical care [11].

Grady et al. (2019) studied a standardized transition program designed to improve outcomes (e.g., adherence to immunosuppression/medical regimen) for young adults who underwent heart transplant as children and transferred to adult care. The program focused on improving heart transplant knowledge, self-care and self-advocacy skills, and enhancing social support. The transition program included computer modules and multiple meetings/telephone calls with dedicated HTx staff. Patients were randomized into the intervention group or to usual care (e.g., standard transfer-of-care meeting). There were no significant between-group or within-group differences in percent of tacrolimus levels within target range from baseline to 6 months (intervention 69–75%, usual care 58–72%) [15]. Additionally, average overall self-reported adherence to the treatment regimen was similarly good in both groups, and no significant group/time interactions were detected. The intervention group actually ended up having significantly more episodes of acute rejection through the 6 months when compared to usual care, though the overall numbers were low (intervention = 5, usual care = 0).

### 3.7. Studies’ Methodological Quality

Table 6 outlines bias ratings for cohort studies, as scored by the Newcastle–Ottawa scale. Table 7 outlines bias ratings for cross-sectional studies, as scored by the modified Newcastle–Ottawa scale. Table 8 outlines the bias rating for the randomized trial, as scored by Version 2 of the Cochrane risk-of-bias tool for randomized trials. The six cohort studies scored between 7 and 9 out of 9 possible points, with points deducted for lack of controls/adjustments and self-reported outcomes. The seven cross-sectional studies scored between 6 and 8 out of 10 possible points, with points deducted for lack of description of non-respondents, lack of controls/adjustments, only self-reported outcomes, and incomplete presentation of measurement of association. The majority of points were deducted for lack of controls/adjustments for cofounders (20 points amongst 11 studies). The randomized trial scored a low overall risk, acknowledging the nonblinded participants and researchers.

## 4. Discussion

This systematic review outlined measures of adherence and baseline adherence estimates in pediatric heart transplant patients. Baseline adherence rates varied greatly, with most values between 40% and 70%, depending on measurement metrics. Adherence was measured most often with serum immunosuppression levels, though the way in which these levels were reported, interpreted, and assigned clinical significance differed between studies. Some studies also used a validated self-report measure. Variable immunosuppressant drug levels correlated with significant clinical outcomes, including rejection, hospitalizations, and death. Mental health comorbidities were associated with nonadherence in some studies. Finally, findings regarding the utility of transition programs in improving adherence in pediatric patients were mixed.

An agreed upon gold standard for measuring adherence would be beneficial for data analysis and identification of at-risk patients. By doing so, we would be better equipped to compare results between studies, especially in a patient population that is already limited in size. Of note, the lack of standardization for measuring adherence is not an issue unique to pediatric transplant recipients. In the adult literature, there are numerous tools to measure immunosuppression adherence [28]. The majority of these tools are self-report measures [28], which have their limitations (including Hawthorne-type effects and social desirability). While many pediatric studies utilized immunosuppressant drug levels to determine adherence, this was less common in the adult literature [28]. It is worth noting that recent efforts led to the development of the PROMIS Medication Adherence Scale (PMAS), which is a widely available, free self-report measure of adherence. Validation studies for PMAS are ongoing to evaluate its psychometric properties in different pediatric and adult patient populations [29].

Our findings are consistent with the literature, illustrating how intrapatient variability of a drug is associated with poor allograft outcomes (e.g., rejection, death) [30,31]. Cardiac allograft vasculopathy (CAV) is a cause of morbidity and mortality in pediatric heart transplant patients, and is a common indication for re-transplantation. Interestingly, the link between medication nonadherence and CAV has not been clearly elucidated. Per the International Society for Heart and Lung Transplantation (ISHLT) data, rejection within the first year is associated with CAV development [32]. However, in adults, tacrolimus intrapatient variability was not associated with cardiac allograft vasculopathy [33]. Pediatric patients with cardiac allograft vasculopathy have a 50% allograft survival rate at nearly three years [34]. A variety of factors can lead to CAV, including rejection [35].

The relationship between medication nonadherence, quality of life and mental health is likely multifactorial. Symptoms of anxiety and depression may make medication adherence more difficult and lead to intentional nonadherence. It is also possible that medication nonadherence may lead to emotional distress and physical symptoms, which may negatively impact quality of life and mental health. Adult heart transplant patients also struggle with mental health and socioeconomic comorbidities, as well as engaging with support networks [28]. When discussing medication adherence, time should be spent addressing a patient’s mental health concerns to identify those that would benefit from psychiatric/psychological intervention.

Lastly, this review showcased the feasibility of two pediatric to adult heart transplant transition programs and their potential to improve immunosuppression adherence. These publications support the continued development of transition programs for these patients, with an opportunity for further research to improve adherence outcomes. In addition to transition programs, there may be a role for technology-based interventions to improve adherence in heart transplant patients. Digital interventions are already being used for children and adolescents with many chronic health conditions [36,37,38,39,40,41,42,43,44,45,46,47]. Gomis-Pastor et al. published one of the first studies using a mobile health intervention (mHeart) to improve immunosuppression adherence in adult heart transplant patients [48]. Use of the app was significantly associated with improved adherence to immunosuppression, increasing adherence rates from 61% to 87% [48]. A recently published systematic review on mobile health app interventions in transplant recipients showed that medication adherence improved in the majority of studies evaluating m-health as an intervention [49]. The continued development of technology-based interventions to improve medication adherence remains a promising area of research, one which may hopefully lead to improved health outcomes and quality of life.

### Strengths and Limitations

The primary strength of this manuscript is its systematic approach to evaluating immunosuppression adherence in pediatric heart transplant patients. All articles were independently screened by at least two authors, and articles were evaluated for bias by two authors. The data were carefully studied and subsequently organized in an attempt to provide clinically relevant information.

One limitation of this paper is the heterogeneous nature of the data. The variety of ways in which immunosuppression levels were described, as well as the combination of different validated self-report measures and qualitative outcomes, precludes a meta-analysis. There is also a lack of controls and adjustments in the data analysis. The included studies have relatively small sample sizes, which is to be expected, given the nature of the pediatric heart transplant cohort. Patient follow-up varied amongst the included studies, limiting the interpretation of timing for outcomes associated with nonadherence. Lastly, only articles published in English were included in our review.

Given that immunosuppressive regimens have changed over the past few decades, comparing data from studies over a 20-year time span has its limitations. The majority of HTx patients are prescribed a three-drug maintenance immunosuppression regimen, including a CNI, antimetabolite, and corticosteroids. Cyclosporine is a CNI that was most popular in clinical practice in the 1980s [50], while tacrolimus was introduced in the early 1990s and has become the preferred CNI due to a more favorable side-effect profile [50]. Additionally, mycophenolate mofetil largely replaced azathioprine as the antimetabolite of choice after the clinical trial by Kobashingawa et al. (1998) showed reduced rates of rejection and improved survival with mycophenolate mofetil [51]. Sirolimus and everolimus are proliferation signal inhibitors that became available in the early 2000s [50]. These drugs may be used as part of a triple-drug regimen or for CNI avoidance, though particular use cases are beyond the scope of this manuscript. There may be a role for simplified medication regimens to improve adherence rates [52,53], which remains a meaningful topic for additional research and review.

## 5. Conclusions

Nonadherence to immunosuppression in pediatric heart transplant remains a challenge that has an impact on patient outcomes (rejection, hospitalization, mortality), quality of life, and mental health. A gold-standard adherence measure would assist with collective analysis and interpretation of the pediatric heart transplant literature. Transition programs are feasible interventions for young adult patients with the aim to improve adherence, but more research is needed to determine associated outcomes. Lastly, further studies are needed to identify additional strategies to improve adherence in pediatric heart transplant patients.

## Figures and Tables

**Table 1:** Characteristics of all included studies.

Author/Year	Population	Study Design and Number of HTx Participants	Participant Age	Measure of Adherence	Duration and Attrition Rate
Albert (2012) [23]	38 patients who received heart transplantation between 1–18 yo and were 16–34 yo at time of participation in Berlin, Germany	Cross sectional, 38	16–34 years	Medication Experience Scale for Immunosuppressants (MESI)	N/A, N/A
Anton (2019) [11]	12 patients in Dallas, TX	Retrospective cohort, 12	16–22 years	Immunosuppressant drug levels, Medical Condition and Medication Knowledge Questionnaire	2 years, N/A
Devine (2011) [12]	Adolescent patients between 11–20 years old who received solid organ transplants (47 kidney, 20 liver, 14 heart, 1 double lung) and parents in Atlanta, GA	Cohort, 14	11–20 years and their parents	The Medication and Clinic Attendance Modules of the Medication Adherence Measure (MAM), Parent and adolescent reports of missing doses or late doses (adherence determined as number of doses taken on time divided by number prescribed), Rescheduled clinic appointments	18 months, 19% (16/82)
Eaton (2018) [13]	47 patients who received solid organ transplant and their caregivers (47 AYA-caregiver dyads, 94 total participants) in Atlanta, GA	Cross sectional, 13	12–19 years	The Medication Adherence Measure (MAM), The Medication Level Variability Index (MLVI) for tacrolimus	N/A, N/A
Flippin (2000) [14]	49 patients in St. Louis, MO	Retrospective cohort, 49	0–18 years	Immunosuppressant drug levels	Follow up ranged from 6–138 months, N/A
Grady (2019) [15]	88 patients at six pediatric heart transplant programs in the USA (East, Midwest and West)	Randomized Controlled Trial, 88	Mean age 21 years with SD 3 years	Immunosuppressant drug levels, The Patient Assessment of Problems with the HT Regimen	4 months, 11.3% (10/88)
Grady (2018) [16]	88 patients at six pediatric heart transplant programs in the USA (East, Midwest and West)	Cross sectional, 88	Mean age 21 years with SD 3 years	Immunosuppressant drug levels, The Patient Assessment of Problems with the HT Regimen	N/A, N/A
Kerr (2020) [17]	138 patients > 12 months out from transplant in Seattle, WA	Retrospective cohort, 138	Mean age 5.6 ± 6.1 years	Immunosuppressant drug levels	Mean 5.5 ± 3.6 years, N/A
McCormick (2019) [18]	30 patients in Ann Arbor, MI	Cross sectional, 30	Median age 17.5 years (15.6–19.2)	Immunosuppressant drug levels, Patient self-reported adherence during clinic visits by answering “how many missed or late immunosuppression doses have you had in the last week”	N/A, N/A
Ringewald (2001) [19]	50 patients in Chicago, IL	Retrospective cohort, 50	For the rejection group, age at heart transplant 8.9 ± 6.1 years. For the nonrejection group, age at transplant 6 ± 6.4 years	Immunosuppressant drug levels and voluntary admission of irregular medication administration. Patients were stratified by episodes of late rejection.	N/A, N/A
Serrano-Ikkos (1998) [22]	53 patients who received heart transplant or heart-lung transplant in London, United Kingdom	Prospective cohort, 29	Mean age 10.2 years, SD 5.0 years	Self-reported adherence, review of patient diaries and cyclosporine levels	Followed for 12 months, N/A
Simons (2009) [6]	82 adolescent solid organ transplant recipients and 68 parent/adolescent dyads in the USA	Cross sectional, 14	11–21 years (mean 15.8, SD 2.4 years)	Immunosuppressant drug levels, Medication Module of the Medication Adherence Measure (MAM), Multidimensional Adherence Classification System (MACS)	N/A, 13.4% (11/82)
Wolfe (2020) [20]	27 patients in Aurora, CO	Cross-sectional, 27	4–18 years old (mean 9.3, SD 3.7 years)	Immunosuppressant drug levels	N/A, N/A
Wray (2006) [21]	50 patients who had undergone heart or heart-lung transplantation in London, United Kingdom	Cross sectional, 33	12.3–24.9 years old (mean 17.87, SD 3.41 years)	Immunosuppressant drug levels	N/A, 20% (10/50)

**Table 2:** Measures of Adherence.

Name	Overview
Medication Experience Scale for Immunosuppressants (MESI) [24]	Seven item self-report questionnaire, total scores between 4 and 33 Higher scores reflect more negative attitudes towards immunosuppressant side effects
The Medication Adherence Measure (MAM) [25]	A semi-structured clinical interview to assess medication adherence Consists of three core modules and several treatment specific modules
The Medication Level Variability Index (MLVI) for tacrolimus [26]	MLVI is calculated as the standard deviation of a set of at least three tacrolimus trough blood levels for each participant A higher MLVI means less consistent medication adherence
The Patient Assessment of Problems with the Heart Transplant Regimen [27]	Measures adherence to 15 components of the medical regimen (including immunosuppressants) Higher scores indicate more adherence
Multidimensional Adherence Classification System (MACS) [6]	A four-category classification system that uses a combination of self-reports and serum immunosuppressant assays Developed in solid organ transplant patients
Medical Condition and Medication Knowledge Questionnaire [11]	Assessment developed by study personnel to track medical knowledge, medication adherence, and self-reported confidence to transition from pediatric to adult health care

**Table 3:** Studies exploring drug levels as a marker for nonadherence and rejection.

Author (Year)	Adherence Measure	Outcomes
Simons (2009) [6]	Immunosuppressant drug levels (Tacrolimus and Cyclosporine)	Standard deviation of tacrolimus levels positively correlated with rejection episodes (r = 0.48, *p* < 0.00), hospitalizations (r = 0.43, *p* < 0.00), and mortality (r = 0.40, *p* < 0.00).
	Medication Module of the Medication Adherence Measure (MAM)	Mortality was significantly correlated with adolescent reports of missed doses (r = 0.31, *p* < 0.05)
	Multidimensional Adherence Classification System (MACS)	Overall rate of nonadherence was 61% (across solid organ transplant groups) Probability of experiencing a rejection episode in the past six months was significantly greater than the expected value for members of the Genuinely Non-adherent group than for members of the other three groups χ^2^(3, *n* = 82) = 14.5, *p* < 0.00
Ringewald (2001) [19]	Immunosuppressant drug levels (Tacrolimus and Cyclosporine) and variability (defined as the ratios of non-therapeutic CSA levels over total number of levels analyzed per patient)	Drug variability increased as the number of rejection episodes grew, and mean drug percent variability was higher in the group with rejection (*p* = 0.04). Patients with no rejection had 10% variability for cyclosporine/tacrolimus, whereas drug variability was 20–25% in patients with >4 episodes of rejection.
	Voluntary admission of irregular medication administration	Admitted nonadherence was not associated with an abnormal CSA concentration at admission for rejection. Two thirds of the patients with late rejection (11 of 15) admitted to nonadherence.
Kerr (2020) [17]	Immunosuppressant drug levels (Tacrolimus and Cyclosporine)	57% of participants had at least one subtherapeutic CNI level. 22% required treatment for rejection within 3 months of a subtherapeutic level. The risk of rejection increased 6.9-fold in the 2 weeks following a subtherapeutic level, and 6.1-fold in the 3 months after presenting with a subtherapeutic level, as compared to time period after a therapeutic level.
Flippin (2000) [14]	Cyclosporine levels and variability (defined as the ratios of non-therapeutic CSA levels over total number of levels analyzed per patient).	8 of 49 patients were defined as the high-variability group. History of non-compliance with the chronic treatment plan was present in 5/8 patients as opposed to 0/41 patients in the low-variability group (*p* < 0.001). The high-variability group had a significantly higher median number of hospitalized days (*p* = 0.036) and experienced significantly higher rates of recurrent rejection (*p* = 0.0003). Patients with high variability had a significantly greater death rate more than 6 months after transplant (*p* = 0.01).

CNI = calcineurin inhibitor, CSA = cyclosporin A.

**Table 4:** Studies exploring impact of nonadherence on quality of life and mental health.

Author/Year	Measure of Adherence	Assessments of QOL and Mental Health	Main Outcomes
Albert (2012) [23]	Medication Experience Scale for Immunosuppressants (MESI)	The Short Form Health Survey (SF-36)	The study identified three risk groups: 51% of patients showed a low non-compliance risk based on MESI value. 29% indicated an increased non-compliance risk based on MESI. 20% belonged to non-compliance high-risk group based on MESI. Patients in high-risk non-compliance group had significantly reduced values on scales Vitality (*p* = 0.20), Mental Health (*p* = 0.035), Mental Component Score (*p* = 0.048), and General Health (*p* = 0.005).
		Giessen Subjective Complaints List (GBB)	High risk non-compliance group had significantly more disorders on the scales cardiac disorders (*p* = 0.020), exhaustion (*p* < 0.001), and epigastric pain (*p* = 0.002), and on the total score of the GBB (*p* = 0.002).
		Health Questionnaire for Children and Young People (KIDSCREEN-27)	No significant differences between the control group and patient group re: psychosomatic disorders
Devine (2011) [12]	The Medication and Clinic Attendance Modules of the Medication Adherence Measure (MAM)	The Child Health Questionnaire-Child Form 87 (CHQ-CF87)	Time 2 parent reported physical HRQOL negatively correlated with presence of rejection episodes in last 6 months (r = −0.41, *p* < 0.01). Time 2 adolescent and parent reported mental health correlated with parent reported adherence (r = 0.31, *p* < 0.05) and (r = 0.30, *p* < 0.05), respectively. Time 2 adolescent reported general health receptions were significantly correlated with adolescent reported adherence (r = 0.3, *p* < 0.05), presence of rejection episode in the last 6 months (r = −0.44, *p* < 0.01), and adolescent reported rescheduled clinic visits (r = 0.43, *p* < 0.01). Time 2 parent reported general health perceptions were significantly correlated with presence of rejection episode in the last 6 months (r = −0.25, *p* < 0.05) and parent reported rescheduled clinic visits (r = −0.30, *p* < 0.05).
		The Child Health Questionnaire-Parent Form 50 (CHQ-PF50)	
	Parent and adolescent reports of missing doses or late doses (adherence determined as number of doses taken on time divided by number prescribed)	Family Environment Scale (FES)	
	Rescheduled clinic appointments	End-Stage Renal Disease Symptom Checklist-Transplant Module (ESRD-SCL)	
Eaton (2018) [13]	The Medication Adherence Measure (MAM)	The Adolescent Medication Barriers Scale (AMBS) contains three factors: -Disease Frustration/Adolescent Issues (DF) -Regimen Adaptation/Cognitive Issues(RA) -Ingestion Issues (II)	Transplant type was unrelated to AYA or caregiver proxy-reported nonadherence 15% of AYAs reported missing one or more doses of antirejection medications in the past week 26% of AYAs reported taking one or more doses of antirejection medications late in the past week 6% of caregivers reported that their AYA missed one of more doses of antirejection medications in the past week 28% of caregivers reported that their AYA took one or more doses of antirejection medications late in the past week AYAs who reported nonadherence to antirejection medications had higher barriers on the AMBAS RA scale (d = −1.10) There were no differences on AYA depression of anxiety symptoms based on AYA or caregiver report of any missed medication doses There were no differences on caregiver emotional distress symptoms based on AYA or caregiver reported missed or late doses of antirejection medications Based on MLVI, 60% of patients were adherent, 40% were nonadherent AYAs with MLVIs > 2 (i.e., nonadherent to tacrolimus) had higher scores on the AMBS DF (*p* < 0.05), II (*p* < 0.01), Total scales (*p* < 0.05) and PMBS II scale (*p* < 0.05)
		Parent Medication Barriers Scale (PMBS) Contains four factors: -Disease Frustration/ Adolescent Issues (DF) -Regimen Adaptation/ Cognitive Issues (RA) -Ingestion Issues (II), -Parent Reminder (PR)	
		The Behavior Assessment System of Children-2nd Edition Self-Report of Personality, Adolescent Version (BASC-2-SRP-A)	
	The Medication Level Variability Index (MLVI) for tacrolimus	The Brief Symptom Inventory-18 (BSI-18)	
McCormick (2019) [18]	Immunosuppression trough levels (a standard deviation of trough levels was calculated)	Generalized anxiety disorder-7 scale (GAD-7) PedsQL 4.0 Generic Core Scales PedsQL 3.0 Cardiac Module Post heart transplant fears questionnaire (PHTF)	93% of patients were on tacrolimus, with a median immunosuppression SD of 1.56 20% of patients reported at least one late dose of immunosuppression in the last week 23% of patients reported moderate to severe anxiety symptoms Moderate or severe generalized anxiety symptoms were significantly associated with patients reporting late or missed immunosuppression doses within the past 2 weeks during clinical interview (*p* = 0.004)
	Self-reported adherence during clinic visits		
Serrano-Ikkos (1998) [22]	Self-reported adherence, review of patient diaries	Camberwell Family Interview Schedule (CFI) Semi-structured psychiatric interview of children Patients’ diaries were checked for medication dosage, pulmonary function measurement, and daily completion	69.8% of patients had good adherence 20.8% had moderate adherence (unsatisfactory diary completion) 9.4% had poor adherence (unsatisfactory cyclosporine levels) Heart transplant patients were more adherent than heart-lung transplant recipients (*p* = 0.01) No significant associations between mental illness in the child and adherence No significant associations between child’s level of psychosocial functioning and adherence
	Cyclosporine levels		
Wolfe (2020) [20]	Immunosuppression trough levels (tacrolimus and cyclosporine)	Wechsler Intelligence Scale for Children (WISC) Wechsler Adult Intelligence Scale (WAIS) Wechsler Adult Intelligence Scale (WPPSI) Wechsler Individual Achievement Test (WIAT) California Verbal Learning Test (CVLT) Behavior Rating Inventory of Executive Function (BRIEF) National Institute of Child Health Questionnaire (NICHQ) Adaptive Behavior Assessment System (ABAS)	Better adherence to immunosuppressant medication (as evidenced by CNI levels) was related to parent-reported attention concerns in the child (*p* = 0.02). The number of rejection episodes was inversely related to long-term memory (*p* = 0.03). Adherence was not related to any other variables. ∙
Wray (2006) [21]	Immunosuppression levels (tacrolimus and cyclosporine)	Beliefs about Medication Questionnaire (BMQ) Perceived Illness Experience (PIE)	17 (43%) patients in total were non-adherent. 11 (28%) of patients were unintentionally non-adherent (based on BMQ) and 7 (18%) were intentionally non-adherent with medications. 6 of the 7 patients were intentionally non-adherent with their immunosuppressive medication (supported by CNI levels showing a marked drop from baseline levels). Intentional nonadherence was significantly associated with depression necessitating psychiatric referral (*p* = 0.03) 11 (28%) of patients reported regularly forgetting to take their medications 8 participants (20%) agreed with or were undecided about the BMQ item “People who take medication should stop it every now and again” and 12 (30%) patients agreed with or were undecided about the statement “My medications are a mystery to me.” All episodes of intentional nonadherence were reported between the ages of 14 and 18 yo.

SD = standard deviation CNI = calcineurin inhibitor QOL = quality-of-life HRQOL = health-related quality of life.

**Table 5:** Studies exploring the effect of a transition program on adherence.

Author (Year)	Measure of Adherence	Transition Program	Main Outcomes
Anton (2019) [11]	Immunosuppression levels (tacrolimus and cyclosporine)	2-year structured transition program to improve patients overall medical knowledge, medication adherence, readiness to transition, and parental perceptions of child’s readiness to transition. Consisted of 7 2-hr sessions.	Statistically significant decrease in percentage of CNI levels out of range prior to beginning the transition program and after completing the transition program (*p* < 0.05).
	Medical Condition and Medication Knowledge Questionnaire		
Grady (2018) [16]	Immunosuppression levels	Only discussed baseline data in this paper	With regards to CNI levels (tacrolimus, cyclosporine, and sirolimus), approximately 70% of participants in both the intervention and usual care arms had blood levels within the target range. For tacrolimus, 68.8% and 71.7% had blood levels within target range in the intervention arm and usual care arm, respectively. For cyclosporine, 74.1% and 70% had blood levels within target range in the intervention arm and usual care arm, respectively. For sirolimus, 71.4% and 66.7% had blood levels within target range in the intervention arm and usual care arm, respectively.
	Self-report (Assessment of Problems with the HT Regimen)		
Grady (2019) [15]	Immunosuppression levels	A standardized tailored transition program focused on increasing HT knowledge, self-care and self-advocacy skills and enhancing social support. It was designed to improve outcomes (i.e., adherence to immunosuppression and the medical regimen) for emerging adults who underwent HT as children and transferred to adult care	There were no significant between-group or within-group differences in percent of tacrolimus levels within target range over time (intervention 69–75%, usual care 72–58% (baseline to 6 months)). Average overall self-reported adherence to the treatment regimen was similarly good in both groups, and no significant group/time interactions were detected. The number of patients with adverse events through 3 and 6 months was low to moderate, with a trend toward more adverse events in the intervention group by 6 months. The rates of keeping clinic and CNI blood-draw appointments were similar in both groups through 3 months and 6 months. The number of episodes of treated acute rejection were low through 3 and 6 months but differed significantly between groups through 6 months (intervention = 5, usual care = 0, *p* = 0.021).
	Self-report (Assessment of Problems with the HT Regimen)		

CNI = calcineurin inhibitor HT = heart transplant.

**Table 6:** Bias ratings of cohort studies via Newcastle–Ottawa scores.

File Name	Representativeness of the Exposed Cohort	Selection of Nonexposed Cohort	Ascertainment of Exposure	Demonstration That Outcome of Interest Was Not Present at the Start of the Study	Comparability	Assessment of Outcome	Length of Follow-Up	Adequacy of Follow-Up	Bias Rating	Bias Reasoning
Anton (2019)	1	1	1	1	0	1	1	1	7 out of 9	No controls/adjustment
Devine (2011)	1	1	1	1	2	0	1	1	8 out of 9	Self-reported outcomes
Flippin (2000)	1	1	1	1	0	1	1	1	7 out of 9	No controls/adjustment
Kerr (2020)	1	1	1	1	2	1	1	1	9 out of 9	N/A
Ringewald (2001)	1	1	1	1	0	1	1	1	7 out of 9	No controls/adjustment
Serrano-Ikkos (1998)	1	1	1	1	0	1	1	1	7 out of 9	No controls/adjustment

N/A: not applicable.

**Table 7:** Bias rating of cross-sectional studies via modified Newcastle–Ottawa scores.

File Name	Representativeness of the Sample	Sample Size	Non-Respondents	Ascertainment of the Exposure (Risk Factor)	Comparability	Assessment of the Outcome	Statistical Test	Bias Rating	Bias Reasoning
Albert (2012)	1	1	0	2	1	1	0	6 out of 10	No description of non-respondents, no controls/adjustments done, self-reported outcomes only, *p* values provided but no confidence intervals
Eaton (2018)	1	1	1	2	0	2	0	7 out of 10	No controls/adjustments done, *p* values provided but no confidence intervals
Grady (2018)	1	1	0	2	0	2	1	7 out of 10	No description of non-respondents, no controls/adjustments done
McCormick (2019)	1	1	1	2	0	2	1	8 out of 10	No controls/adjustments done
Simons (2008)	1	1	0	2	0	2	0	6 out of 10	No description of non-respondents, no controls/adjustments done, *p* values not provided
Wolfe (2019)	1	1	0	2	1	2	1	8 out of 10	No description of non-respondents, incomplete controls/adjustments
Wray (2006)	1	1	1	2	0	2	0	7 out of 10	No controls/adjustments done, *p* values not consistently provided

**Table 8:** Bias rating of randomized control trails via version 2 of the Cochrane risk-of-bias tool.

File Name	Randomization Process	Effect of Assignment	Missing Outcome Data	Outcome Measurement	Reported Result	Overall Risk
Grady (2019)	*Low risk* 1.1: Used computer based block randomization 1:3: No differences between intervention groups	*Low risk* 2.1, 2.2: Nonblinded to participants and researchers 2.3: No deviations occurred 2.6: Appears that intention-to-treat was done	*Low risk* 3.1: Nearly all participants completed the study	*Some concerns* 4.1/4.2: Measurement was appropriate and did not differ between groups 4.3/4.4: Assessors were aware of intervention and allocation 4.5: Seems unlikely outcome was affected by knowledge of intervention	*Low risk* 5.1/5.2/5.3: One pre-specified analysis of the data	*Low risk*

## Data Availability

Not applicable.

## Appendix A. Search Strategy

*PubMed*

((“Heart Transplantation” [MeSH Terms] OR “heart transplant*” [Title/Abstract]) OR ((“Organ Transplantation” [MeSH Terms:noexp] OR “transplant *” [Title/Abstract]) AND (“heart” [MeSH Terms] OR “heart *” [Title/Abstract]))) AND (((((((((“Medication Adherence” [MeSH Terms] OR “Patient Compliance” [MeSH Terms]) OR “Adherence” [Title/Abstract]) OR “Nonadherence” [Title/Abstract]) OR “non adherence” [Title/Abstract]) OR “non adherence” [Title/Abstract]) OR “Compliance” [Title/Abstract]) OR “Noncompliance” [Title/Abstract]) OR “non compliance” [Title/Abstract]) OR “non compliance” [Title/Abstract])

Remove review [pt] and animals in title.

Embase

(‘heart transplantation’/exp OR ‘heart transplant*’:ti,ab OR ((‘organ transplantation’/exp OR transplant*:ti,ab) AND (‘heart’/exp OR heart:ti,ab))) AND (‘medication compliance’/exp OR ‘adherence’:ti,ab OR ‘nonadherence’:ti,ab OR ‘non adherence’:ti,ab OR ‘compliance’:ti,ab OR ‘noncompliance’:ti,ab OR ‘non compliance’:ti,ab)

Remove review and meeting abstracts and animals in title.

Central

#1 MeSH descriptor: [Heart Transplantation] explode all trees
#2 “heart transplant*”:ti,ab
#3 #1 OR #2
#4 MeSH descriptor: [Organ Transplantation] explode all trees
#5 transplant*:ti,ab
#6 MeSH descriptor: [Heart] explode all trees
#7 heart:ti,ab
#8 #4 OR #5
#9 #6 OR #7
#10 #8 AND #9
#11 #3 OR #10
#12 MeSH descriptor: [Medication Adherence] explode all trees
#13 MeSH descriptor: [Patient Compliance] explode all trees
#14 (“adherence” OR “nonadherence” OR “non adherence” OR “compliance” OR “noncompliance” OR “non compliance”).ti,ab

Scopus

TITLE-ABS-KEY (“heart transplant*”) AND TITLE-ABS-KEY (adherence OR nonadherence OR “non adherence” OR compliance OR noncompliance OR “non compliance”) AND (LIMIT-TO (DOCTYPE, “ar”))
